# Randomized Trial of Erythromycin and Azithromycin for Treatment of Chlamydial Infection in Pregnancy

**DOI:** 10.1155/S1064744995000718

**Published:** 1995

**Authors:** Marc F. Rosenn, George A. Macones, Neil S. Silverman

**Affiliations:** 1 Department of Obstetrics and Gynecology Division of Maternal-Fetal Medicine Thomas Jefferson University Philadelphia PA USA; 2 Center for Clinical Epidemiology and Biostatistics University of Pennsylvania School of Medicine Philadelphia PA USA; 3 Department of Obstetrics and Gynecology Abington Hospital 1235 Old York Road, Suite 119 Levy Medical Plaza Abington PA 19001 USA

## Abstract

*Objective:* The purpose of this study was to compare erythromycin and azithromycin in the treatment of chlamydial cervicitis during pregnancy with regard to efficacy, side effects, and compliance.

*Methods:* In a prospective manner, 48 pregnant patients with cervical chlamydial infections diagnosed by routine screening tests were randomly assigned to receive either erythromycin, 500 mg q.i.d. for 7 days (N = 24), or azithromycin, 1 g as a one-time dose (N = 24). All sexual partners were given prescriptions for doxycycline, 100 mg b.i.d. for 7 days. The treatment efficacy was assessed by follow-up chlamydia testing 3 weeks after the therapy was completed. The side effects, intolerance to therapy, and overall compliance were evaluated by means of a standardized posttreatment questionnaire.

*Results:* There was no significant difference in cure rates noted between the erythromycin group and the azithromycin group (77% vs. 91%, respectively; *P* = 0.24). Gastrointestinal side effects were reported more frequently among patients treated with erythromycin compared with patients treated with azithromycin (45% vs. 17%, respectively; *P* = 0.004). The patients who received erythromycin reported intolerance to therapy secondary to side effects more frequently than patients who received azithromycin (23% vs. 4%, respectively; *P* = 0.07). Furthermore, the patients in the azithromycin group were more likely to complete their course of therapy as prescribed than the patients in the erythromycin group (100% vs. 61%, respectively; *P* = 0.002).

*Conclusions:* Azithromycin is efficacious and well tolerated for the treatment of chlamydial cervicitis in pregnancy. Erythromycin, though efficacious, is poorly tolerated, as demonstrated by the number of patients reporting significant side effects during the course of therapy. Since the cost of azithromycin is comparable to that of generic erythromycin, the present study supports the use of azithromycin as an alternative to erythromycin for the treatment of chlamydial cervicitis in pregnancy.


Infectious Diseases in Obstetrics and Gynecology 3:241–244 (1995)
© 1996 Wiley-Liss, Inc.

### Randomized Trial of Erythromycin and Azithromycin for Treatment of Chlamydial Infection in Pregnancy

**Marc F. Rosenn, George A. Macones, and Neil S. Silverman**

*Department of Obstetrics and Gynecology, Division of Maternal-Fetal Medicine, Thomas Jefferson University, (M.F.R., G.A.M., N.S.S.); Center for Clinical Epidemiology and Biostatistics, University of Pennsylvania School of Medicine, Philadelphia, PA (G.A.M.)*

### Abstract

*Objective:* The purpose of this study was to compare erythromycin and azithromycin in the treatment of chlamydial cervicitis during pregnancy with regard to efficacy, side effects, and compliance.

*Methods:* In a prospective manner, 48 pregnant patients with cervical chlamydial infections diagnosed by routine screening tests were randomly assigned to receive either erythromycin, 500 mg q.i.d. for 7 days (N = 24), or azithromycin, 1 g as a one-time dose (N = 24). All sexual partners were given prescriptions for doxycycline, 100 mg b.i.d. for 7 days. The treatment efficacy was assessed by follow-up chlamydia testing 3 weeks after the therapy was completed. The side effects, intolerance to therapy, and overall compliance were evaluated by means of a standardized post-treatment questionnaire.

*Results:* There was no significant difference in cure rates noted between the erythromycin group and the azithromycin group (77% vs. 91%, respectively; $P = 0.24$). Gastrointestinal side effects were reported more frequently among patients treated with erythromycin compared with patients treated with azithromycin (45% vs. 17%, respectively; $P = 0.004$). The patients who received erythromycin reported intolerance to therapy secondary to side effects more frequently than patients who received azithromycin (23% vs. 4%, respectively; $P = 0.07$). Furthermore, the patients in the azithromycin group were more likely to complete their course of therapy as prescribed than the patients in the erythromycin group (100% vs. 61%, respectively; $P = 0.002$).

*Conclusions:* Azithromycin is efficacious and well tolerated for the treatment of chlamydial cervicitis in pregnancy. Erythromycin, though efficacious, is poorly tolerated, as demonstrated by the number of patients reporting significant side effects during the course of therapy. Since the cost of azithromycin is comparable to that of generic erythromycin, the present study supports the use of azithromycin as an alternative to erythromycin for the treatment of chlamydial cervicitis in pregnancy. © 1996 Wiley-Liss, Inc.

Key words

Perinatal infection, sexually transmitted diseases, antibiotic therapy, cervicitis

*Chlamydia trachomatis* is the most common sexually transmitted bacterial infection in the United States.[1] The reported prevalence of this infection among pregnant women ranges from 5% to 26%, depending on the population studied.[2] Chlamydial infections have been associated in some studies with adverse perinatal outcomes such as low birth weight because of preterm rupture of the membranes and subsequent preterm delivery.[3–6] Neonatal infection is manifested by clinical conjunctivitis in 20–35% and neonatal pneumonia in 10–20% of infants born to mothers with untreated chlamydial cervicitis.[7,8] The diagnosis and treatment of chlamydia-infected women before delivery

Address correpondence/reprint requests to Dr. Marc F. Rosenn, whose current address is Department of Obstetrics and Gynecology, Abington Hospital, 1235 Old York Road, Suite 119, Levy Medical Plaza, Abington, PA 19001.

Received November 1, 1995

Accepted February 29, 1996

have been shown to significantly lower the risk of these perinatal and neonatal complications.[9–11]

The current first-line treatment for chlamydial infection in pregnant women as recommended by the Centers for Disease Control and Prevention (CDC) is erythromycin base, 500 mg q.i.d. for 7 days.[12] Erythromycin, however, is commonly associated with gastrointestinal side effects which often prevent patients from completing the prescribed course of therapy. Consequently, the failure rate of erythromycin in pregnant women has been reported to be as high as 15%.[9] Alternative antibiotics, such as amoxicillin, have been shown to be equally effective as erythromycin but better tolerated.[13,14] Azithromycin, which has been shown to be as effective as standard therapy for the treatment of chlamydia in nonpregnant patients, has the advantage of being a one-time dose regimen.[15,16]

This randomized trial was conducted to evaluate the efficacy of azithromycin as an alternative to erythromycin for the treatment of chlamydial infections during pregnancy. We also sought to compare the rates of side effects and compliance of the 2 regimens.

### SUBJECTS AND METHODS

All pregnant women registering for care at Thomas Jefferson University Hospital's prenatal clinic undergo cervical chlamydia screening at their first prenatal visit. From August 1994 through April 1995, all prenatal patients with positive cervical chlamydia screening tests were offered enrollment in this study. The exclusion criteria included first prenatal visit at ≥36 weeks, antibiotic use for any indication within 14 days of enrollment, diagnosed coinfection with *Neisseria gonorrhoeae,* and known allergy or sensitivity to either of the study medications.

All cervical specimens were obtained from the endocervical canal, after the ectocervix was cleared of secretions, with a Dacron-tipped swab. The specimens were transported to the virology laboratory for analysis with a commerically available test kit for detection of chlamydia-specific DNA sequences using polymerase chain reaction (PCR) amplification (Amplicor STD Specimen Collection and Transport Kit, Roche Diagnostic Systems, Nutley, NJ). The swabs were transported and stored in Amplicor™ specimen transport media until processing; all specimens not evaluated within 24 h of collection were stored in a −75°C freezer until analysis. We have previously demonstrated this assay to have a sensitivity for chlamydia detection that is superior to standard McCoy cell culture (97% vs. 88%).[17] In this study, the samples that were positive by PCR but negative by culture were subsequently culture-positive with repeat testing or spin-down procedures. In addition, the positive predictive values of the 2 testing techniques were found to be equivalent (98% for PCR and 100% for culture).

Each eligible patient gave written informed consent in accordance with a protocol approved by the University's Institutional Review Board. They were randomly assigned to 1 of 2 treatment groups: erythromycin base, 500 mg orally, q.i.d. for 7 days, or azithromycin, 1 g orally as a one-time dose. The patients in both groups were supplied with medication at the time of enrollment. Their sexual partners were all given prescriptions for doxycycline, 100 mg orally b.i.d. for 7 days. The patients were instructed to abstain from sexual intercourse until after their follow-up appointment.

The treatment group assignment was determined through block-of-six randomization generated from a random-number table. The treatment regimens were placed within sequentially numbered, sealed opaque envelopes, with the clinic staff involved in patient enrollment unaware of the randomization block size.

All patients were scheduled for follow-up appointments for retesting 3 weeks after completing their assigned therapy. They were instructed to report if they or their sexual partners were unable to complete the prescribed course of antibiotics and to record the reason for their noncompliance. Compliance was defined as a completion of the course of therapy as prescribed. Each patient was given a 1-page side effect questionnaire at the time of enrollment which was collected during their return visit for a follow-up test-of-cure.

Normally distributed continuous variables were compared between the groups by means of an unpaired Student's t-test. Ordinal measurements as well as non-normally distributed continuous variables were compared with Wilcoxon's rank sum test. $P < 0.05$ was considered significant. All reported comparisons were 2-tailed.

### RESULTS

A total of 48 eligible patients were enrolled in the study and randomized to 1 of the 2 treatment arms.

TABLE 1. Patient demographics

| | Erythromycin | Azithromycin | P |
|---|---|---|---|
| Mean age (years) | 19.8 ± 3.3 | 21.3 ± 4.0 | 0.17 |
| Mean weight (lb) | 155 ± 29 | 162 ± 30 | >0.5 |
| Mean gestational age at enrollment (weeks) | 19.5 ± 4.5 | 19.3 ± 3.5 | >0.5 |

TABLE 2. Cure rate, side effects, and compliance

| | % Azithromycin (N = 23) | % Erythromycin (N = 22) | P |
|---|---|---|---|
| Cure rate | 91 | 77 | 0.24 |
| Side effects (all gastrointestinal) | 17 | 45 | 0.004 |
| Intolerance to therapy | 4 | 23 | 0.07 |
| Compliance | 100 | 61 | 0.002 |

Only 1 patient declined entry. She was treated with erythromycin, but not included in this analysis.

The demographic characteristics of the patients in each treatment group are shown in Table 1. There were no statistically significant differences in age, parity, weight, or gestational age at the time of enrollment between the 2 groups.

Of the 48 patients, 24 received azithromycin and 24 received erythromycin. Three patients were lost to follow-up, 1 in the azithromycin group and 2 in the erythromycin group. The overall cure rates were 91% for the azithromycin group and 77% for the erythromycin group (Table 2). This difference was not statistically significant.

The treatment groups were compared with regard to side effects, intolerance to therapy, and overall compliance (Table 2). Gastrointestinal side effects were reported by 17% of patients in the azithromycin group compared with 45% of the patients in the erythromycin group ($P = 0.004$). Intolerance to therapy due to severe gastrointestinal side effects was reported by 4% of the patients in the azithromycin group compared with 23% of the patients in the erythromycin group ($P = 0.07$). Three patients in the erythromycin group did not take the medication as prescribed, although their noncompliance was unrelated to side effects. These patients acknowledged not always remembering to take their medication at appropriately close intervals and therefore skipped several doses. Consequently, the overall compliance was noted to be 100% in the azithromycin group compared with 61% in the erythromycin group ($P = 0.002$).

### DISCUSSION

The current standard therapy for a pregnant woman with a cervical chlamydial infection is erythromycin base, 500 mg q.i.d. for 7 days.[12] Because of the high rate of gastrointestinal side effects with this treatment, many patients are unable to complete the prescribed course of therapy. This decrease in patient compliance results in a decrease in efficacy and a concomitantly lower impact on the prevention of both perinatal and neonatal effects of untreated maternal chlamydial infections.

Several studies have evaluated alternative treatment regimens for antepartum chlamydia using medications that are equally effective but better tolerated than erythromycin. Amoxicillin has been shown to be equally efficacious to erythromycin in the treatment of chlamydia in pregnancy, with significantly lower rates of side effects.[13,14] Still, amoxicillin requires treatment over an extended time which may result in noncompliance. It has been demonstrated, in fact, that the level of compliance with prescribed therapies deteriorates with longer courses of tablet taking.[18]

Azithromycin is the prototype of a new group of antibiotics known as the azalides. It is chemically related to erythromycin, but differs in its microbiologic spectrum, tolerability, and unique pharmacokinetics. It is avidly taken up by cells, resulting in high and sustained concentrations in tissues. This unique pharmacokinetic profile allows it to be effective when administered as a one-time dose.

In vitro studies have shown that azithromycin has excellent activity against clinical isolates of *C. trachomatis*.[19] Clinical experience with azithromycin in non-pregnant patients has shown it to be as effective as "standard" therapy for the treatment of chlamydial cervicitis.[15,16] It is FDA approved as a class B medication for use during pregnancy. However, limited experience exists with regard to the efficacy of a single-dose drug such as azithromycin in pregnant patients, who exhibit drug metabolism and plasma volumes of distribution quite different from nonpregnant women. The cost of azithromycin tablets is roughly twice that of generic erythromycin. The new azithromycin powder preparation, however, is comparable in cost with generic erythromycin.

Only 1 series has been published to date comparing azithromycin with erythromycin for the treatment of chlamydial infections in pregnant women. In a randomized study, Bush and Rosa[20] reported a 100% cure rate for azithromycin compared with a 93% cure rate for erythromycin, with significantly fewer side effects among the patients receiving azithromycin. This study, however, had a sample size of 30 patients which limited its ability to detect a meaningful difference in treatment effect.

The results of our study suggest that azithromycin is as efficacious as erythromycin for the treatment of chlamydial infections in pregnancy. It is true that the possibility of a $\beta$ or type II error in our study exists in view of the small sample size. For example, in order to detect a difference in treatment effect between the groups of 50% (assuming an $\alpha$ error = 0.05, a $\beta$ error = 0.2, and baseline failure rate in the control group of 10%), we estimated that approximately 472 patients would be required in each group. Therefore, our results provide preliminary evidence that azithromycin may be a reasonable efficacious alternative treatment for infection with *C. trachomatis* during pregnancy.

### REFERENCES

1. Centers for Disease Control: Recommendations for the prevention and management of *Chlamydia trachomatis* infections. 1993. MMWR 42(RR-12):1, 1993.
2. Stamm WE, Holmes KK: *Chlamydia trachomatis* infections of the adult. In Holmes KK, Mardh PA, Sparling PF, Wiesner PJ (eds): Sexually Transmitted Diseases. 2nd ed. New York: McGraw-Hill, p 181, 1990.
3. Harrison HR, Alexander ER, Weinstein L, Lewis M, Nash M, Sim DA: Cervical *Chlamydia trachomatis* and mycoplasmal infections in pregnancy. Epidemiology and outcomes. JAMA 250:1721, 1983.
4. Martin DJ, Koutsky L, Eschenbach DA, et al.: Prematurity and perinatal mortality in pregnancies complicated by maternal *Chlamydia trachomatis* infections. JAMA 247:1585, 1982.
5. Gravett MG, Nelson HP, DeRouen T, Critchlow C, Eschenbach DA, Holmes KK: Independent association of bacterial vaginosis and *Chlamydia trachomatis* infection with adverse pregnancy outcome. JAMA 256:1899, 1986.
6. Sweet RL, Landers DV, Walker C, Schachter J: *Chlamydia trachomatis* infection and pregnancy outcome. Am J Obstet Gynecol 156:824, 1987.
7. Schachter J, Grossman M, Sweet RL, Holt J, Jordan C, Bishop E: Prospective study of perinatal transmission of *Chlamydia trachomatis.* JAMA 255:3374, 1986.
8. Harrison HR, English MF, Lee CK, Alexander ER: *Chlamydia trachomatis* infant pneumonitis: Comparison with matched controls and other infant pneumonia. N Engl J Med 288:702, 1978.
9. Schachter J, Sweet RL, Grossman M, Landers D, Robbie M, Bishop E: Experience with the routine use of erythromycin for chlamydial infections in pregnancy. N Engl J Med 314:276, 1986.
10. Ryan GM, Abdella TN, McNeeley G, Baselski VS, Drummond DE: *Chlamydia trachomatis* in pregnancy and effect of treatment on outcome. Am J Obstet Gynecol 162:34, 1990.
11. McGregor JA, French JI: *Chlamydia trachomatis* infection during pregnancy. Am J Obstet Gynecol 164:1782, 1991.
12. Centers for Disease Control: 1993 Sexually transmitted diseases treatment guidelines. MMWR 42(RR-14):1, 1993.
13. Silverman NS, Sullivan M, Hochman M, Womack M, Jungkind DL: A randomized, prospective trial comparing amoxicillin and erythromycin for the treatment of *Chlamydia trachomatis* in pregnancy. Am J Obstet Gynecol 170:829, 1994.
14. Alary M, Joly JR, Moutquin J-M, Mondor M, et al.: Randomized comparison of amoxicillin and erythromycin in treatment of genital chlamydial infection in pregnancy. Lancet 344:1461, 1994.
15. Steingrrimson O, Olafsson JH, Thorarinsson H, Ryan RW, Johnson RB, Tilton RC: Azithromycin in the treatment of sexually transmitted disease. J Antimicrob Chemother 25(Suppl A):109, 1990.
16. Martin DH, Mroczkowski TF, Dalu ZA, et al.: A controlled trial of a single dose of azithromycin for the treatment of chlamydial urethritis and cervicitis. N Engl J Med 327:921, 1992.
17. Bass CA, Jungkind DL, Silverman NS, Bondi JM: Clinical evaluation of a new polymerase chain reaction assay for detection of *Chlamydia trachomatis* in cervical specimens. J Clin Microbiol 31:2648, 1993.
18. Cramer JA, Scheyer RD, Mattson RH: Compliance declines between clinic visits. Arch Intern Med 150: 1509, 1990.
19. Scieux C, Kappus EW, Quinn TC: In vitro activity of azithromycin against *Chlamydia trachomatis.* J Antimicrob Chemother 25(Suppl A):7, 1990.
20. Bush MR, Rosa C: Azithromycin and erythromycin in the treatment of cervical chlamydial infection during pregnancy. Obstet Gynecol 84:61, 1994.
